# Responses of Litter Decomposition and Nutrient Dynamics to Nitrogen Addition in Temperate Shrublands of North China

**DOI:** 10.3389/fpls.2020.618675

**Published:** 2021-01-20

**Authors:** Jianhua Zhang, He Li, Hufang Zhang, Hong Zhang, Zhiyao Tang

**Affiliations:** ^1^Department of Biology, Xinzhou Teachers University, Xinzhou, China; ^2^Department of Geographical Sciences, School of Geography, Geomatics and Planning, Jiangsu Normal University, Xuzhou, China; ^3^College of Environment and Resource, Shanxi University, Taiyuan, China; ^4^Department of Ecology, College of Urban and Environmental Sciences, and Key Lab for Earth Surface Processes of the Ministry of Education, Peking University, Beijing, China

**Keywords:** litter decomposition, nutrient dynamics, N deposition, temperate shrublands, North China

## Abstract

Plant litter decomposition is a crucial ecosystem process that regulates nutrient cycling, soil fertility, and plant productivity and is strongly influenced by increased nitrogen (N) deposition. However, the effects of exogenous N input on litter decomposition are still poorly understood, especially in temperate shrublands, which hinders predictions of soil C and nutrient dynamics under the context of global change. Temperate shrub ecosystems are usually N-limited and particularly sensitive to changes in exogenous N input. To investigate the responses of *Vitex negundo* and *Spiraea trilobata* litter decomposition to N addition, we conducted a field experiment in *Vitex*- and *Spiraea*-dominated shrublands located on Mt. Dongling in Beijing, North China. Four N treatment levels were applied: control (N_0_; no N addition), low N (N_1_; 20 kg⋅N⋅ha^–1^⋅year^–1^), moderate N (N_2_; 50 kg⋅N⋅ha^–1^⋅year^–1^), and high N (N_3_; 100 kg⋅N⋅ha^–1^⋅year^–1^). The litter decomposition in *V. negundo* was faster than that in *S. trilobata*, which may be due to the differences in their nutrient content and C/N ratio. N addition increased the amount of remaining N in the two litter types but had no effect on the remaining mass, C, or P. Nitrogen treatment did not affect the litter decomposition rates (*k*) of either litter type; i.e., N addition had no effect on litter decomposition in temperate shrublands. The neutral effect of N addition on litter decomposition may be primarily explained by the low temperatures and P limitation at the site as well as the opposing effects of the exogenous inorganic N, whereby exogenous N inhibits lignin degradation but promotes the decomposition of readily decomposed litter components. These results suggest that short-term N deposition may have a significant impact on N cycling but not C or P cycling in such shrub ecosystems.

## Introduction

Litter decomposition plays a key role in the global carbon (C) and nitrogen (N) balance (Vivanco and Austin, 2011) and is an important process that controls C and nutrient cycling in most terrestrial ecosystems (Mo et al., 2004). Litter decomposition is a result of the combined effects of interactive chemical, biological, and physical processes (Aerts, 2006). The decomposition rate and nutrient release pattern are dependent on abiotic factors (e.g., climate conditions and soil physical and chemical properties) and biotic factors (e.g., litter quality and microbial community composition) (Prescott, 2005; Yu et al., 2015; Song et al., 2018; Lin et al., 2019). At the global scale, plant litter decomposition is controlled by climate and litter quality (Sun et al., 2004; Cornwell et al., 2008; Zhang et al., 2008); at the local scale, it is regulated by litter chemical properties and site soil conditions (Zhang et al., 2016; Zhu W. Y. et al., 2016; Ren et al., 2018).

Anthropogenic N inputs to terrestrial ecosystems through atmospheric N deposition have enhanced soil N availability (Galloway and Cowling, 2002; Galloway et al., 2008; Liu et al., 2013) and have been shown to affect litter decomposition (Mo et al., 2006; Liu et al., 2010; Frey et al., 2014). The site-specific ambient N deposition rate, the background N status, and the litter quality are the most important factors controlling the response of litter decomposition to N addition (Knorr et al., 2005). N input causes a direct increase in soil nutrient availability that may indirectly influence litter quality by altering plant growth, carbon and nutrient allocation patterns in plant tissues, leaf nutrient resorption, and plant stoichiometry (Minocha et al., 2000; Zhang et al., 2015; Zhou et al., 2017). N input may change the components of litter during the decomposition progress (Zhu X. M. et al., 2016). N fertilization may also change the soil pH and/or influence decomposer community, microbial, and soil enzyme activity (Carreiro et al., 2000; Chen et al., 2013; Lu et al., 2014; Yu et al., 2015), which also affect litter decomposition.

Numerous studies in various ecosystems have been conducted to explore the effects of N deposition on litter decomposition (Tu et al., 2014; Yu et al., 2015; Zhu W. Y. et al., 2016; Zhu X. M. et al., 2016; Wang et al., 2017; Ren et al., 2018; Yan et al., 2019; Pei et al., 2020; Zhang et al., 2020). However, the responses of litter decomposition to N addition have varied considerably, from positive (Downs et al., 1996; Allison et al., 2009; Hobbie et al., 2012; Ren et al., 2018) or neutral (Prescott, 1995; Hobbie and Vitouesk, 2000) to negative (Micks et al., 2004; Hobbie et al., 2012; Zhou et al., 2017). Some chemical, biological, and biochemical hypotheses have been proposed to explain the possible mechanisms of the different effects of N deposition on litter decomposition, but the effects of N input on litter decomposition are still poorly understood (Carreiro et al., 2000; Knorr et al., 2005; Liu et al., 2010; Prescott, 2010; Sinsabaugh, 2010; Lu et al., 2014).

Shrub communities usually develop in nutrient-poor sites (Gorissen et al., 2004; Wessel et al., 2004), which are often considered to be relatively sensitive to environmental changes such as atmospheric N deposition (Bobbink et al., 1998; Zhang et al., 2015). To date, there have been few reports about the effect of N addition on the process of litter decomposition in shrub ecosystems. The lack of research on this subject severely limits the accurate assessment of soil C sequestration under increased N deposition, which will lead to a poor understanding of nutrient cycling in shrub ecosystems. To evaluate the effects of increasing N deposition on the litter decomposition process in temperate shrublands of northern China, we conducted a field litter decomposition experiment in two representative shrub communities over a 23-month period. Previous studies have suggested that total N, C/N, C/P, lignin, and cellulose are the main litter quality variables (Tu et al., 2014; Zhu X. M. et al., 2016; Zhou et al., 2017) regulating litter decomposition and nutrient release (Månsso and Falkengren-Grerup, 2003; Zhu W. Y. et al., 2016). Litters with low C/N ratios and higher endogenous N concentrations result in a faster dissolution rate (Su et al., 2004). N or P content in litters often fails to meet the demands of the decomposers, resulting in the immobilization of nutrients in litter at the initiation of decomposition (Gosz et al., 1973). Zhu X. M. et al. (2016) found that N deposition will decrease litter N release when litter decomposition is limited by insufficient N. Moreover, it has been proposed that in N-limited ecosystems, the litter generally lacks N; in addition, N becomes the main factor limiting microbial activity, exogenous N input meets the microbial demand for this element (Deng et al., 2007), and the added N stimulates microbial activity (Fog, 1988; Neff et al., 2002; Fang et al., 2007). Exogenous N also increases enzyme activity (Keeler et al., 2009) or contributes to the production of high-quality litters (Suding et al., 2005), all of which lead to an increase in decomposition. Ren et al. (2018) found that simulated N deposition stimulated litter decomposition in a desert steppe under nutrient-deficient conditions. Thus, we proposed the following hypotheses: (1) N addition would stimulate litter mass loss in the temperate shrublands of northern China, (2) N addition would inhibit litter nutrient release during the early decomposition stage, and (3) the decomposition rate of high-quality litters is faster than that of low-quality litters.

## Materials and Methods

### Site Description and Experimental Design

The study was conducted on Mt. Dongling (39°48′ N–40°02′ N latitude, 115°24′ E–115°36′ E longitude) in Beijing, North China. The study area has a temperate continental monsoon climate, with a warm, humid summer and dry, cold winter. The mean annual temperature (MAT) is 6.3°C, with a minimum temperature of −10.1°C in January and a maximum temperature of 18.3°C in July. The mean annual precipitation is approximately 612 mm, with most falling between June and August. The soil at the study site is classified as a cinnamon soil according to the Chinese Soil Taxonomy (RGCST, 2001). The estimated atmospheric inorganic wet N deposition at this site was approximately 14.5 kg⋅ha^–1^⋅year^–1^ (Zhang et al., 2015). We performed our study in *Vitex negundo* and *Spiraea trilobata* shrublands, and the general information about the shrublands is shown in Table 1.

**TABLE 1:** Elevation, mean annual temperature (MAT), soil total C (STC), soil total N (STN), and soil total P (STP) of the experimental sites (from Zhang et al., 2015).

Community type	Elevation (m)	MAT (°C)	pH	STN (mg⋅g^–1^)	STC (mg⋅g^–1^)	STP (mg⋅g^–1^)
*Vitex negundo*	791	8.2	8.7	2.52^*a*^ (0.33)	26.36^*a*^ (4.38)	0.50^*a*^ (0.05)
*Spiraea trilobata*	1,170	6.4	8.9	2.20^*a*^ (0.24)	34.71^*a*^ (5.49)	0.50^*a*^ (0.02)

*Values are expressed as the mean of three samples with the standard error (SE) in parentheses. Data in the same column followed by the same letters are not significantly different (Tukey’s multiple-comparison test; *P* > 0.05).*

In each community, four treatments with three replicates each were established in a randomized block design in twelve 5 m× 5 m plots at intervals of 3 m in the shrub ecosystems in June 2012. Four N deposition levels were established for this study: control (N_0_; without N addition), low N deposition (N_1_: 20 kg⋅N⋅ha^–1^⋅year^–1^), moderate N deposition (N_2_: 50 kg⋅N⋅ha^–1^⋅year^–1^), and high N deposition (N_3_: 100 kg⋅N⋅ha^–1^⋅year^–1^). The N was added as CO(NH_2_)_2_. The fertilizer was applied in five equal monthly doses from May to September. N fertilizer was sprayed from July to September in the first year (2012) and then from May to September afterward (2013–2014). At each application, the fertilizer was dissolved in 2 L of water and sprayed evenly back and forth at a height of 10 cm above the ground in each plot. The control plots received 2 L of water without fertilizer at each application time.

In late October 2012, freshly fallen *V. negundo* and *S. trilobata* leaf litters were collected from non-treated shrub communities of each species outside of the experimental blocks. After the litter was air-dried to a constant weight, 5 g of litter from each species was placed into nylon-mesh litter bags (size of 10 cm × 15 cm, with an upper layer of 2-mm mesh and a lower layer of 0.3-mm mesh). Five litterbags for each species were randomly selected and brought to the laboratory to determine the initial chemical properties of the litter (Table 2). On November 20, 2012, these litterbags were then placed in the treatment plots of their respective species. In each plot, 30 bags (8 sampling dates × 3 bags per sampling time + 6 bags to account for unexpected losses), for a total of 360 litterbags, were fixed to the ground surface with metal pins to prevent them from moving. Three bags were collected from each plot at eight time points, i.e., at the end of May, June, July, August, and September 2013 and of May, July, and October 2014. After retrieval, the remaining litter was removed from the bag and carefully cleaned of soil and other extraneous materials. The remaining litter in each bag was oven dried at 65°C for 48 h, weighed, and then milled to measure its C, N, and P concentrations.

**TABLE 2:** Initial chemical properties of the leaf litter.

Litter type	N (mg⋅g^–1^)	C (mg⋅g^–1^)	P (mg⋅g^–1^)	C/N	C/P	N/P
*Vitex negundo*	11.05 (0.31)a	465.56 (1.17)a	0.52 (0.01)a	42.27 (1.20)a	890.92 (21.34)a	21.10 (0.41)a
*Spiraea trilobata*	8.74 (0.17)b	466.29 (3.89)a	0.36 (0.02)b	53.42 (1.82)b	1,315.17 (64.43)b	24.66 (1.35)b

*Values are the mean, and the SE is in parentheses; *n* = 5 for all samples. Data in the same column followed by different letters are significantly different (*P* < 0.05).*

### Chemical Analyses

The total C and N concentrations of the leaf materials were measured using an elemental analyzer (2400 IICHNS/O, Perkin-Elmer, United States) (Zhang et al., 2015), and the total P concentration was measured by a molybdate/ascorbic acid method after H_2_SO_4_–HClO_4_ digestion (John, 1970).

### Data Analysis

We used a power function to fit the decomposition processes (Olson, 1963):

(1) $$\begin{array}{c} y=a\times e{}^{\left(-kt\right)} \end{array}$$

where *y* is the remaining mass (%) at time *t* (years), *k* is the decomposition coefficient (year^–1^), and *a* is the correction factor. The percentage remaining (*R*) of biomass and element content (C, N, and P) for each period (*X*_*i*_) were determined and compared with the initial values (*X*_0_) following Zhou et al. (2017):

(2) %R=(X/iX)0×100

The time to 50% (*T*_50_%) decomposition of the litter samples was estimated based on the *k* values following Bockheim et al. (1991):

(3) T%50=ln(1-0.50)/(-k)

A simple *T*-test was used to determine the differences in initial litter chemical properties between the two litter species. One-way analysis of variance (ANOVA) with the least significant difference (LSD) test was used to evaluate the significant differences in the mass and element content (C, N, and P) remaining among N treatments and the control (CK) for each sampling date. A repeated-measures ANOVA with Tukey’s honestly significant difference (HSD) test was performed to examine the overall effect of N addition on the remaining mass; C, N, and P; and the C/N and C/P ratios of the decomposing leaf litter. The *k* values of the incubation periods were calculated based on replicates within each block, and ANOVA was also performed to test for differences in the decomposition rate constant (*k*) and *T*_50_% among N addition treatments. Two-way ANOVA was used to test for the main and interactive effects of N addition and decomposition time on the remaining biomass and element content (C, N, and P) as well as on the C/N ratio and C/P ratio.

Statistical analysis was conducted in SPSS 17.0 (Norusis, 2009).

## Results

### Initial Chemical Quality of Leaf Litter

At the beginning of the experiment, the N and P concentrations in the leaf litter of *V. negundo* were significantly higher, but C/N, C/P, and N/P were lower than those of *S. trilobata* (*P* < 0.05). However, there was no significant difference in C concentrations between the two leaf litters (*P* > 0.05).

### Effects of N Addition on the Decomposition Rate

**FIGURE 1:**
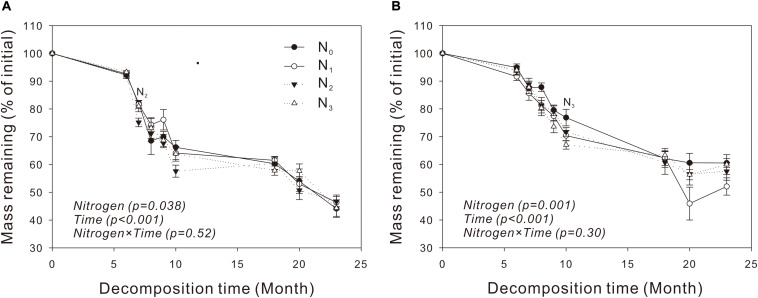
Dynamics of the remaining mass in leaf litter of *Vitex negundo* **(A)** and *Spiraea trilobata* **(B)** at different N addition levels during the whole experimental period. Text in italics indicates the effects of N and time, as well as their combined effects, based on the two-way ANOVA. N_1_, N_2_, and N_3_ at each decomposition time indicate that the difference between the specified N treatments and the control (CK) was significant (*P* < 0.05). Error bars represent the standard deviations of the means (*n* = 3). N_0_, control; N_1_, low N; N_2_, moderate N; and N_3_, high N.

For both species, the percentage of litter remained relatively stable during the cold and dry seasons (November to April) and decreased rapidly in the warm and wet seasons (May to September) (Figures 1A,B). The amount of mass in the two litters decreased exponentially with time in the N treatment plots (*P* = 0.038, *P* = 0.001, respectively), and N_2_ and N_3_ increased significantly litter decomposition in *V. negundo* and *S. trilobata* after 7 and 10 months, respectively (Figures 1A,B). A repeated-measures ANOVA showed that N addition did not influence the amount of remaining mass in the litter samples (*P* > 0.05, Table 3). Furthermore, none of the treatments had an impact on the *T*_50_% or the decomposition rate constant *k* (Table 4), indicating that although N treatment promoted decomposition of two litters from two temperate shrublands in the early decomposition stage, it had no significant influence on the total or average decomposition rate of litters. In addition, we found that litter decomposition in *V. negundo* was faster than that in *S. trilobata* in all treatments during the whole decomposition period (Figures 1A,B and Tables 3, 4).

**TABLE 3:** Overall effects of N addition on the mass; the remaining C, N, and P; and the C/N and C/P ratios (mean ± SE) of decomposing foliar litter of *Vitex negundo* and *Spiraea trilobata* in northern China.

Treatment	Remaining (% of initial)				C/N	C/P
	Mass	C	N	P		
***Vitex negundo***						
N_0_	67.25.4*a*	57.006.54*a*	87.043.97*a*	100.335.05*a*	26.962.03*a*	501.1744.09*a*
N_1_	68.75.4*a*	57.876.87*a*	92.905.18*b*	101.905.44*a*	25.701.90*ab*	500.6739.22*a*
N_2_	65.25.2*a*	56.576.15*a*	94.684.34*b*	95.805.74*a*	24.771.69*b*	522.3335.32*a*
N_3_	67.55.4*a*	58.986.34*a*	101.325.01*c*	99.355.76*a*	24.111.71*b*	523.4934.97*a*
***Spiraea trilobata***						
N_0_	76.264.85*a*	69.025.73*a*	97.762.11*a*	104.925.07*a*	37.722.53*a*	882.4077.83*a*
N_1_	70.853.44*a*	64.955.93*a*	98.572.28*a*	101.544.75*a*	34.862.56*b*	836.2859.04*a*
N_2_	73.725.10*a*	66.845.56*a*	100.732.04*b*	103.553.85*a*	34.182.46*b*	848.2655.06*a*
N_3_	72.794.79*a*	67.635.46*a*	108.921.57*c*	99.913.44*a*	33.062.42*b*	894.7456.33*a*

*Each column represents the mean value across all sampling dates in all treatments. Different letters indicate significant differences among different treatments in each community (repeated-measures ANOVA with Tukey’s HSD test, α = 0.05, *n* = 3). N_0_, control; N_1_, low N; N_2_, moderate N; and N_3_, high N.*

**TABLE 4:** The decomposition rate constant (*k*) and the time to 50% (*T*_50__%_) decomposition in the different treatments.

Shrubland type	Treatment	Decomposition rate constant (*k*) (year^–1^)	Coefficient of determination (*r*^2^)	Time to 50% (*T*_50__%_) decomposition (years)
*Vitex negundo*	N_0_	0.40 ± 0.00a	0.79	1.73 ± 0.01a
	N_1_	0.39 ± 0.02a	0.84	1.80 ± 0.08a
	N_2_	0.41 ± 0.03a	0.82	1.69 ± 0.11a
	N_3_	0.40 ± 0.02a	0.81	1.76 ± 0.09a
*Spiraea trilobata*	N_0_	0.30 ± 0.02a	0.83	2.36 ± 0.19a
	N_1_	0.39 ± 0.06a	0.80	1.87 ± 0.29a
	N_2_	0.33 ± 0.06a	0.80	2.15 ± 0.20a
	N_3_	0.32 ± 0.03a	0.79	2.19 ± 0.12a

*Values are means with 1 ± SE; *n* = 3. The same lowercase letters in the same row indicate a non-significant difference between different treatments at the 0.95 confidence level. *r*^2^: Coefficient of determination of negative exponential decomposition equations of litter decomposition. N_0_, control; N_1_, low N; N_2_, moderate N; and N_3_, high N.*

### Effects of N Addition on C, N, and P Release

**FIGURE 2:**
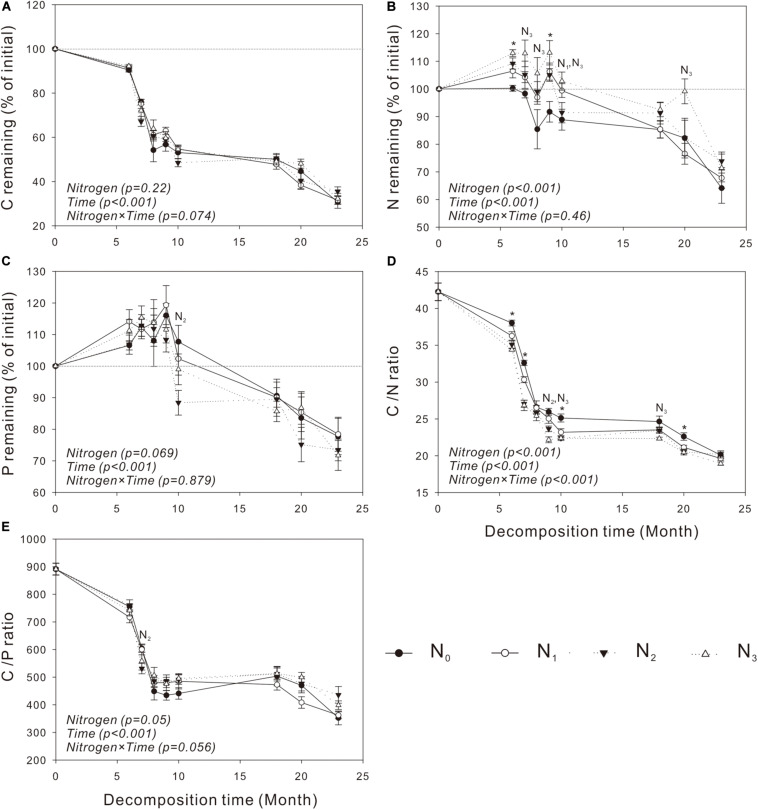
Dynamics of the remaining C, N, and P and the C/N and C/P ratios in *Vitex negundo* leaf litter **(A–E)** at different N addition levels during the whole experimental period. Text in italics indicates the effects of N and time, as well as their combined effects, based on the two-way ANOVA. N_1_, N_2_, and N_3_ at each decomposition time indicate that the difference between the specified N treatments and the control (CK) was significant (*P* < 0.05). The symbol * means that all N treatments were significantly different from the control. Error bars represent the standard deviations of the means (*n* = 3). N_0_, control; N_1_, low N; N_2_, moderate N; and N_3_, high N.

**FIGURE 3:**
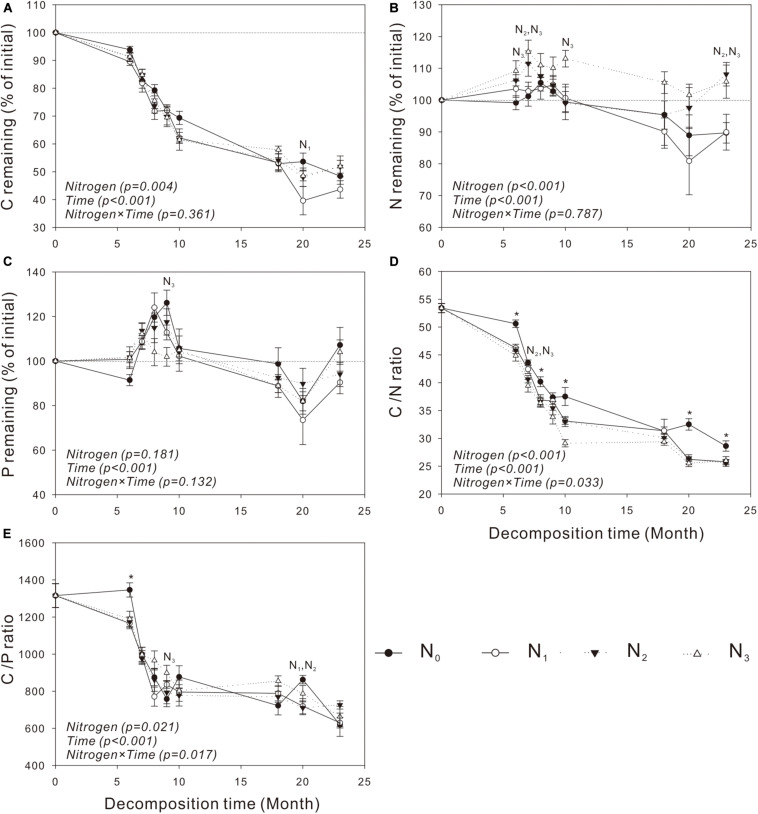
Dynamics of the remaining C, N, and P and the C/N and C/P ratios in leaf litter of *Spiraea trilobata* **(A–E)** at different N addition levels during the whole experimental period. Text in italics indicates the effects of N and time, as well as their combined effects, based on the two-way ANOVA. N_1_, N_2_, and N_3_ at each decomposition time indicate that the difference between the specified N treatments and the control (CK) was significant (*P* < 0.05). The symbol * means that all N treatments were significantly different from the control. Error bars represent the standard deviations of the means (*n* = 3). N_0_, control; N_1_, low N; N_2_, moderate N; and N_3_, high N.

Similar to mass loss, C release was generally very slow in the cold and dry seasons but quite fast in the warm and wet seasons (Figures 2A, 3A). The N treatments significantly affected C release from *S. trilobata* litter (*P* = 0.004, Figure 3A), whereas they had negligible effects on C release from *V. negundo* litter (*P* = 0.22, Figure 2A). The remaining N and P in *V. negundo* litter increased mainly in the early stage of decomposition and decreased in the later stage of decomposition, while those in *S. trilobata* litter increased mainly in the early stage before decreasing and later increasing again in the later stage of decomposition. The highest values of remaining N were generally observed in the N_3_ plots of the *V. negundo* and *S. trilobata* shrublands (Figures 2B,C, 3B,C). N addition significantly increased the amount of remaining N in *V. negundo* and *S. trilobata* litter compared with that in the control (*P* < 0.05 for both; Table 3 and Figures 2B, 3B), whereas it exerted no impact on the remaining P (*P* > 0.05 for both; Table 3 and Figures 2C, 3C).

## Discussion

### Response of Litter Decomposition to N Addition

In our study, we found that N addition significantly increased litter decomposition of *V. negundo* and *S. trilobata* in the early decomposition process (Figures 1A,B), but it affected neither the *k* values nor the mass loss (i.e., overall or average decomposition rate) of the two litters (Tables 3, 4). These results indicated that N treatment did not affect litter decomposition of these two species, which did not support our first hypothesis. Similar results were also found by Fang et al. (2007) and Tu et al. (2014), and they found that although litters decomposed faster in the N treatment plots in certain months, N treatment did not affect litter decomposition (Fang et al., 2007; Tu et al., 2014). Our results are also consistent with the results of Zhu W. Y. et al. (2016), who found that N addition did not significantly affect the decomposition of litter of any of the four investigated species after 2 years of N fertilization in an alpine meadow on the Qinghai-Tibet Plateau. However, our results differed from those of Han et al. (2014), who found that nitrogen deposition inhibited temperate forest litter decomposition in a *Quercus liaotungensis* forest that is subject to chronically high levels of ambient N deposition. Moreover, Ren et al. (2018) found that nitrogen addition significantly promoted the decomposition of *Stipa breviflora* litter in a desert steppe.

There are several explanations for the independence of litter decomposition from N addition. First, if the litter or its environment itself is not deficient in N, increasing N will not have a significant influence on litter decomposition (Kwabiah et al., 1999). Second, the decomposition-accelerating effect of the increased N on the more labile carbon fractions may have been offset by the inhibiting effect of N on lignin decay (Fog, 1988; Prescott, 1995). Third, the quality of the C sources in litter affects the response of decomposers to exogenous N (Hobbie and Vitouesk, 2000). In our study, N addition depressed the remaining mass in certain months in both communities, although not significantly (Figures 1A,B), probably due to the inhibition of lignin degradation by N in the later stages of decomposition (Conn and Day, 1996; Berg and McClaugherty, 2008; Zhou et al., 2017). This inhibition process can counteract the decomposition-promoting effect of N on the labile carbon fractions, leading to the decomposition rate in our study not corresponding to the nitrogen addition level. It may also be due to the variation in decomposition rates between sampling dates and between treatments (Tu et al., 2014). In addition, the MAT at our study site (6.3°C) was lower than the decomposition threshold value (10°C), which can inhibit the overall decay process (Prescott, 2010; Zhu W. Y. et al., 2016; Figures 1A,B). The rate of decomposition in the initial stage (December 2012 to May 2013) was very slow, likely because litter decomposition was limited by the low temperatures at that time; this resulted in less mass loss during these months than in other periods of the year (Figures 1A,B). Approximately half of the incubation time occurs during the cold season (Supplementary Figure 1); therefore, the low decomposition rate during the cold months in our study seriously affected the overall decomposition rate.

The N/P ratio in green leaves has been demonstrated to be a valid indicator of nutrient limitation, with values >16 indicating P limitation (Koerselman and Meuleman, 1996). The higher N/P ratios (>16) and extremely high C/P ratios observed for litter in this study may indicate a greater shortage of P than of N (Table 2). We also found that the N/P ratio (18.4) of all dominant species in our research site was considerably higher than 16, indicating P limitation but not N limitation in the two shrublands of Mt. Dongling; thus, the relative shortage of P might also be an important control on litter decomposition in these two temperate shrublands. P limitation may contribute to explaining why the decomposition rate was not affected by N addition in our study.

Consistent with our third hypothesis, we found that *V. negundo* litter decomposed faster than *S. trilobata* litter in all treatments during the whole decomposition period (Figures 1A,B and Tables 3, 4). This result may be due to differences in the chemical composition of the litters (Table 2), e.g., their nutrient content and C/N, C/P, and N/P ratios. This idea is supported by a recent meta-analysis reporting that litter quality parameters (such as the C/N, C/P, N/P, and lignin/N ratios) and total nutrient content control litter decomposition rates, even at the global scale (Valenzuela-Solano and Crohn, 2006; Cornwell et al., 2008; Prescott, 2010; Gong et al., 2020).

### Dynamics of Litter C, N, and P Under Different N Addition Treatments

As the primary energy source for litter decomposers (Ågren and Bosatta, 1996; Manzoni et al., 2010), litter C is the main constituent of the total litter mass. Thus, it is not surprising that the amount of litter C decreased continuously during the whole decomposition period (Figures 2A, 3A); similar results have been obtained in studies of other ecosystems (Zhang and Wang, 2012; Peng et al., 2014; Zhou et al., 2017). Litter nutrient dynamics can be used to reflect nutrient availability to the decomposer community (Hobbie, 2005). Because of the high levels of N and P relative to C in decomposers, the content of N or P in litter is often insufficient for the decomposers, resulting in the immobilization of nutrients in litter at the initial stage of decomposition (Gosz et al., 1973). N and P are released from litter only when the needs of the decomposers are met (Yang et al., 2017). In our study, the net immobilization of N and P mainly occurred in the early stage of the decomposition of the two litters. In the late decomposition stage, *V. negundo* showed a net release of N and P, while the *S. trilobata* litter showed net release, net immobilization, or release followed by immobilization. The accumulation of litter N in the present study may be due to the activity of decomposer organisms that import N from the soil solution to the decomposing litter (Parton et al., 2007; Berglund and Ågren, 2012) to meet their need for N. Generally, more N tends to be retained in litter in plots with high levels of N addition (Berg and Tamm, 1994; Apolinário et al., 2013), and this phenomenon was observed in the present study (Figures 2B, 3B and Table 3). In our study, we also found that N addition exerted no impact on the remaining P (Table 3 and Figures 2C, 3C) (partly consistent with our second hypothesis), which is consistent with the result of Zheng et al. (2017).

The net N release and immobilization were strongly controlled by the initial C/N ratio of the leaf litter (Parton et al., 2007). Some studies found that a net N release occurs only when the average C/N ratio of the litter is less than 40 (Parton et al., 2007). In our study, the C/N ratio of *V. negundo* and *S. trilobata* litter was higher than 40 (Table 2 and Figures 2D, 3D); thus, net N immobilization occurred during the experiment (Figures 2B, 3B). Considering that N deposition may affect the leaf nutrient resorption proficiency (i.e., the N concentration of the leaf litter) (Soudzilovskaia et al., 2007), in the future, increased N deposition in this region may change not only the pattern but also the magnitude of initial N releases. The pattern of P release was strongly controlled by the C/P ratio of the litter (Zhu W. Y. et al., 2016). Gosz et al. (1973) and Dziadowiec (1987) found that the critical C/P values for P release are in the range of 200 to 480, which is far lower than those in our study (Table 2 and Figures 2E, 3E). Similarly, net P immobilization occurred during our experiment (Figures 2C, 3C). Our results are similar to those of other studies reporting that N inputs can cause microbial nutrient retention without having a simultaneous impact on the mass loss of the decomposing litter (Cleveland et al., 2006; Mo et al., 2008).

## Conclusion

In conclusion, N addition had no effect on the remaining mass, C, or P in leaf litter but significantly suppressed N release in temperate shrublands, indicating that short-term N deposition has a significant impact on N cycling but not on C or P cycling in such shrub ecosystems. N addition had no significant effects on the decomposition of leaf litter. This may have been due to low temperatures and P limitation at this site or to the opposing effects of the exogenous inorganic N. These opposing effects may have masked the stimulating effects of nitrogen in some months. The decomposition rate and N and P dynamics were controlled strongly by the initial C/N and C/P ratios of the leaf materials. The different phases of leaf litter decomposition were clearly governed by different limiting factors. These results provide an important data basis for the simulation and prediction of C and nutrient cycle processes in temperate shrub ecosystems under scenarios of increasing N deposition. However, due to the lack of knowledge regarding the impacts of these processes on microbial communities, soil enzyme activities, and soil fauna, the results of this 23-month experiment are not suitable for predicting the long-term status of litter decomposition and C and N cycles in such ecosystems. Thus, further investigations are needed to fully understand the effects of long-term N deposition on litter decomposition and nutrient release in the studied shrub ecosystems.

## Data Availability Statement

The datasets presented in this study can be found in online repositories. The names of the repository/repositories and accession number(s) can be found in the article/Supplementary Material.

## Author Contributions

ZT conceived and designed the experiments. JZ and HL performed the experiments. JZ analyzed the data. JZ and ZT wrote the manuscript. All the authors made a substantial, direct, and intellectual contribution to this work.

## Conflict of Interest

The authors declare that the research was conducted in the absence of any commercial or financial relationships that could be construed as a potential conflict of interest.

## Supplementary Material

Supplementary Figure 1 Mean monthly air temperatures and monthly precipitation during 1993 to 2010 from the meteorological station adjacent to study site.

Supplementary Dataset 1 Mass, C, N, and P concentrations in leaf litter of *Vitex negundo* and *Spiraea trilobata* across all sampling dates in all treatments.
