# Supplementary material for: Responses of Litter Decomposition and Nutrient Dynamics to Nitrogen Addition in Temperate Shrublands of North China
Source: Front Plant Sci. 2021 Jan 20;11:618675. doi: 10.3389/fpls.2020.618675 (PMC7873982; doi:10.3389/fpls.2020.618675)
Supplement: Supplementary Figure 1 — Mean monthly air temperatures and monthly precipitation during 1993 to 2010 from the meteorological station adjacent to study site. [file Data_Sheet_1.docx]

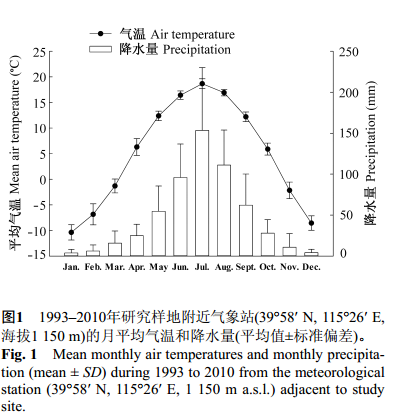


Figure S1 Mean monthly air temperatures and monthly precipitation (mean ± SD) during 1993 to 2010 from the meteorological station (39°58′ N, 115°26′ E, 1 150 m a.s.l.) adjacent to study site (Su et al., 2012).

Su, H. X., Bai, F., and Li G. Q. (2012). Seasonal dynamics in leaf area index in three typical temperate montane forests of China: a comparison of multi-observation methods. Chinese Journal of Plant Ecology, 2012, 36, 231-242. doi: 10.3724/SP.J.1258.2012.00231
